# Editorial: Sensory Hair Cell Death and Regeneration

**DOI:** 10.3389/fncel.2016.00208

**Published:** 2016-08-31

**Authors:** Michael E. Smith, Andrew K. Groves, Allison B. Coffin

**Affiliations:** ^1^Department of Biology, Western Kentucky UniversityBowling Green, KY, USA; ^2^Departments of Neuroscience and Molecular and Human Genetics, Baylor College of MedicineHouston, TX, USA; ^3^Departments of Integrative Physiology and Neuroscience, Washington State UniversityVancouver, WA, USA

**Keywords:** hair cells, auditory, regeneration, cell death, ototoxicity, hearing loss, otic development, cochlea

Sensory hair cells are specialized mechanosensory receptors found in vertebrate auditory, vestibular and lateral line organs that transduce vibratory and acoustic stimuli into the sensations of hearing and balance. Sensorineural hearing loss typically occurs when hair cells are damaged from infection, noise exposure, drugs (called ototoxins), and age-related decline. Unfortunately, as hair cell regeneration does not occur to any significant extent in mammals, damage to these cells in humans leads to hearing and balance disorders. The signaling mechanisms that cause hair cell damage are incompletely understood and vary with the type of damage involved, and a given form of damage can activate multiple cell signaling cascades, complicating our understanding of these damage processes and the development of potential therapeutics. Yang et al. present a comprehensive overview of hair cell damage across stimuli. Other papers in this Research Topic offer more focused reviews or original research, as described below.

## Damage and protection

Several papers in this Frontiers Research Topic address cell signaling that underlies hair cell damage from a variety of sources. Van Rossom et al. investigated programmed cell death resulting from variants in DFNA5, a gene tightly linked to autosomal dominant hearing loss in human families. DFNA5 is located on chromosome 7 and multiple variants are correlated with deafness in different human families. Using yeast, Van Rossom et al. demonstrate that truncated *DFNA*_*5*_ (*mutDFNA*_*5*_) alters expression of mitochondrial and endoplasmic reticulum (ER)-related genes, while *mutDFNA*_*5*_ expression in HEK293 cells up-regulated expression of MAPK signaling members. *MutDFNA*_*5*_ expression increased phospho-JNK expression in HEK cells and reduced cell viability, which could be rescued by pharmacological inhibition of JNK signaling. This suggests that *mutDFNA*_*5*_ induces cell death via oxidative stress mechanisms associated with mitochondrial function, and perhaps via ER stress, via MAPK signaling.

While genetic causes of hearing loss accounts for the majority of congenital deafness, ototoxin exposure can cause hearing loss later in life. Aminoglycoside antibiotics are used to treat a wide-variety of bacterial infections, and up to 25% of patients receiving aminoglycosides suffer significant hearing loss. Aminoglycoside ototoxicity is extensively studied, because these antibiotics are well characterized, relatively simple to administer in animal models, and produce dose-dependent sensory tissue lesions. Multiple papers in this Research Topic investigate aspects of aminoglycoside-induced hair cell death and protection.

Tao and Segil used RNA-Seq to profile transcriptional changes in hair cells exposed to the aminoglycoside gentamicin. They cultured neonatal cochleae from Atoh1-GFP transgenic mice, then purified GFP+ hair cells for sequencing and bioinformatics analysis. A 3 h exposure to gentamicin changed gene expression in several cell death and survival pathways, particularly JNK and NF-kB, and in several cell cycle checkpoint and proliferation genes. The effects on these latter pathways are interesting because hair cells are post-mitotic, and these data suggest that gentamicin treatment may disrupt cell cycle control. As aminoglycosides also cause nephrotoxicity, Tao and Segil then compared their results with those from researchers who studied gentamicin-treated kidney cells and found similar changes in gene expression across multiple gene classes. Their results suggest that gentamicin-mediated damage in hair cells causes changes in JNK and NF-kB signaling that precedes cellular stress responses and programmed cell death.

Sun et al. investigated cell death and survival genes in mouse cochleae treated with the aminoglycoside neomycin and found increased expression of pro-cell death genes such as *Diablo, Htra2*, and multiple caspases. Interestingly, these changes were most pronounced in young animals (P8-P14), implying a “sensitive period” in which cochlear hair cells are more susceptible to damage. Using a transgenic mouse line that over-expresses the pro-survival gene *Xiap*, Sun et al. demonstrated that increased *Xiap* expression reduced caspase levels in cochlear hair cells and conferred some protection from neomycin-induced hearing loss.

Oxidative stress is a key mediator of hair cell death, with multiple studies reporting increased reactive oxygen species (ROS) following aminoglycoside exposure and antioxidant-related hair cell protection. Dinh et al. summarize several of these ROS studies, as well as highlighting the complexity of hair cell death signaling overall, including activation of both classical apoptotic and non-apoptotic components. Majumder et al. examined one component of the oxidative stress response, the ROS scavenger glutathione (GSH), during cochlear development and neomycin exposure. Using a fluorescent reporter, they showed that GSH levels increase with age in the mouse cochlea and that GSH expression does not change across the length of the cochlea, which is surprising as basal hair cells are more susceptible to damage than apical hair cells. Even more surprising, pharmacologic depletion of GSH did not sensitize cochlear hair cells to neomycin damage. These results add to the ongoing debate about the role of oxidative stress in aminoglycoside ototoxicity.

Other than mammalian models, zebrafish, a common model organism to study vertebrate development and gene function, is also used to study hair cell death and regeneration. Like other non-mammalian vertebrates, zebrafish have the capacity to regenerate lost sensory hair cells (reviewed in Monroe et al.). The zebrafish lateral line is particularly useful for screening genes and compounds that affect hair cell responses to ototoxin exposure. Stawicki et al. review several of these studies, describing 3 novel mutations and over 20 compounds associated with reduced aminoglycoside susceptibility. Uribe et al. used the zebrafish lateral line as a model system to determine if pharmacologic activation of hepatocyte growth factor (HGF) signaling conferred protection from aminoglycoside toxicity. The HGF mimetic Dihexa protected hair cells from neomycin damage via activation of c-Met, the cognate HGF receptor, and conferred protection via Akt and MEK signaling.

Li et al. examined the interplay between aminoglycoside ototoxicity and acoustic over-exposure, a “double-whammy” that causes increased hair cell damage (Li et al.). Prior studies demonstrated that mechanotransduction (MET) channels at the stereociliary tips are the primary route of aminoglycoside entry into hair cells (Alharazneh et al., 2011; Vu et al., 2013). Li et al. show that in mice, exposure to wide-band noise increases gentamicin uptake in outer hair cells. Increased gentamicin uptake was associated with a decrease in tip links, a seemingly counter-intuitive finding, because a reduction in tip links should reduce MET channel opening and therefore reduce aminoglycoside entry. Li et al. suggest that the absence of functional mechanotransduction in some hair cells increases aminoglycoside entry into remaining functional cells, or that hair cells have multiple aminoglycoside uptake routes, including some that are independent of transduction such as TRPA1. While the mechanism of enhanced aminoglycoside uptake remains largely unknown, Li et al. demonstrate that moderately loud sound exposure enhances uptake, resulting in increased outer hair cell loss.

## Development and regeneration

The hair cells of the mature mammalian cochlea are terminally differentiated and once lost cannot be regenerated from other cell types. Examining the process of development of sensory hair cells in mammals, and comparing the pathways involved in regeneration in non-mammalian vertebrates, has been a source of potential molecular targets to induce hair cell regeneration in mammals. Macrophages are implicated in the development, regeneration, and repair of numerous tissues (Wynn et al., 2013). Kaur et al. show that macrophages are recruited to areas of hair cell injury in the mouse utricle and actively phagocytose hair cell debris as a precursor to hair cell regeneration. Fractalkine, a chemokine that plays a role in the interaction between the immune and nervous systems, can release a diffusible fragment that can attract macrophages. Using a diphtheria toxin model to kill hair cells, Kaur et al. found that fractalkine was not required for macrophage entry into the damaged tissue, suggesting that macrophage recruitment is mediated by other signaling factors.

One way to look for genes likely important in regulating hair cell regeneration is by examining genes that are important in the process of early ear development. Hartman et al. used microarray analysis to find genes specifically upregulated in the early mouse ear and found sensory lineage-specific transcripts that included *Fbxo2, Col9a2*, and *Oc90*. These genes were also significantly up-regulated in both hair cells and supporting cells, suggesting that they could be good sensory lineage markers and might advance developmental and regeneration studies.

The Notch and Wnt pathways have been extensively studied in hair cell development and regeneration (Murata et al., 2012). Jansson et al. contribute a detailed review of Wnt signaling in relationship to hair cell development and regeneration, demonstrating the complex roles of Wnt signaling in inner ear patterning, cell proliferation, and cell type specification. Two papers in this Research Topic focus on Notch signaling, a key signaling pathway regulating cell fate decisions in the inner ear. Maass et al. show that mouse cochlear supporting cells lose their ability to trans-differentiate into hair cells by the time mice are six days old, even when the Notch pathway is blocked. This result is explained in part by a down-regulation of Notch receptors and ligands at this stage. A known regulator of Notch activity in *Drosophilia* is Numb, which is thought to regulate asymmetric cell division and thus differentiation. Eddison et al. overexpressed Numb at different stages in the developing chick inner ear. Surprisingly, they found that Numb did not significantly repress Notch activity in the inner ear.

Knowledge gained from the role played by Wnt and Notch signaling in hair cell development has led researchers to investigate how the process of hair cell regeneration recapitulates developmental pathways. Two downstream target genes of the Wnt pathway are highlighted in this Research Topic: *Lgr5* and *Lgr6*. Lgr5 and Lgr6 are members of the leucine-rich repeat-containing G-protein-coupled receptors (LGRs). Lin et al. found that Lgr5 was expressed in a subset of supporting cells in the striolar region of the neonatal mouse utricle following neomycin-induced hair cell loss. Further, they showed that Lgr5-positive supporting cells can regenerate new hair cells in explant cultures. Zhang et al. characterized the spatiotemporal expression of Lgr6 in the cochlear duct of embryonic and postnatal mice. They found that Lgr6 was first expressed in a single row of prosensory cells in the middle and basal turn of the cochlea, followed by expression being restricted to inner pillar cells (IPCs), and then the inner border cells (IBCs), after which expression disappeared. Wnt signaling was required to maintain Lgr6 expression *in vitro* and Lgr6-positive cells had the capability to differentiate into hair cells in culture. Thus, Lgr5- and/or Lgr6-postive cells may serve as hair cell progenitors in the mammalian cochlea.

Advances in biotechnology are improving ways that researchers can study molecular mechanisms that function in hair cell death and regeneration. For example, Tarang et al. recently developed a tetracycline-inducible TetO-CB-myc6-Rb1 (CBRb) transgenic mouse model to achieve transient and inducible dominant-negative inhibition of the protein retinoblastoma 1 (RB1), an endogenous protein essential in regulating cellular proliferation, differentiation, and homeostasis. Down-regulation of RB1 leads to increased proliferation and supernumerary hair cells in the cochlea, suggesting that RB1 may be a therapeutic target.

It is unlikely that the manipulation of one gene or the administration of a single compound will promote mammalian hair cell regeneration because (1) hair cell regeneration proceeds via multiple cellular pathways, and (2) epigenetic regulation controls these pathways. In support of point 1, Jahan et al. review the plethora of genes involved in hair cell proliferation and differentiation in relation to the key transcription factor Atoh1, suggesting that a more nuanced view of Atoh1 regulation is necessary to explain potentially conflicting data. As support for point 2, chromatin remodeling, by histone modifications such as acetylation, has been shown to be important in cellular processes such as development and regeneration. Histone deactylases (HDACs) remove acetyl groups from histone tails, which represses transcription. Following hair cell ablation with neomycin, pharmacological inhibition of HDACs increased histone acetylation in zebrafish lateral line hair cells and decreased the number of proliferating supporting and regenerated hair cells (He et al.). The underlying mechanism of HDAC regulation of hair cell regeneration is unknown, but pathways involved in HDAC or histone acetyltransferases (HATs) may be targets for therapeutics promoting hair cell regeneration. In support of this point, Layman and Zuo offer a review of epigenetic regulation in the inner ear in relationship to development, otoprotection, and hair cell regeneration.

## Future directions

Collectively, these papers highlight the recent progress in understanding the cellular and molecular processes involved in sensory hair cell death and regeneration. While there currently is no cure for sensorineural hearing loss in humans, recent experiments like those highlighted in this Research Topic provide a clearer picture of what genes and signaling mechanisms are involved and what therapies may prevent hearing loss, and potentially promote hair cell regeneration in the future.

Of course, there are numerous questions still left unanswered. For example, what are the relative advantages and disadvantages of using stem cells as opposed to genetic manipulations of *in situ* supporting cells to induce transdifferentiation into new hair cells? While the use of stem cell-derived precursor cells show promise, technical difficulties include maintaining the survival of cell grafts in the proper locations along the cochlea without further damaging the cochlea, prevention of tumor formation, and potential graft-vs.-host rejection. Transdifferentiation of *in situ* supporting cells into hair cells is also difficult, as supporting hair cells represent a terminally differentiated state that is not overly amenable to manipulation. Since there are numerous molecular pathways involved in the development of sensory hair cells, researchers are not likely to find a single silver bullet in the form of a specific gene or protein that will restore hearing when manipulated or supplied therapeutically. Although supernumerary new hair cells have been induced in the mature mammalian cochlea, this has rarely led to any improvement in hearing function due to the precise organization of the rows of hair cells in the organ of Corti and the necessity of forming synaptic contacts between these hair cells and the appropriate spiral ganglion neurons (Hayashi et al., 2007; Gubbels et al., 2008; Mansour et al., 2009; but see Izumikawa et al., 2005 for an example of improved sensitivity in auditory evoked potentials).

As a result of these difficulties in restoring lost sensory hair cells, more researchers are focusing on ways to prevent hair cell loss in the first place. For example, a number of the studies in this research topic focused on ototoxin-induced cell death pathways and compounds that can mitigate such hair cell loss (He et al.; Li et al.; Majumder et al.; Stawicki et al.; Sun et al.; Tao and Segil; Uribe et al.). So far, this work has centered around a few ototoxic compounds such as the aminoglycosides gentamicin or neomycin. Since different ototoxic compounds can have different effects on hair cells, much more research is needed to get a more holistic view of how to prevent ototoxin-induced hair cell loss. For example, some evidence suggests that aminoglycoside antibiotics enter hair cells via the mechanotransduction channel (Marcotti et al., 2005; Alharazneh et al., 2011; Vu et al., 2013), suggesting that temporary channel block may attenuate ototoxic damage by reducing toxin uptake. However, this strategy may not be appropriate for other ototoxins, and indeed inhibition of intracellular signaling may be preferable in all cases, as a temporary channel block would reduce auditory function. Future work is needed to establish the best therapeutic approaches for each ototoxin.

## Author contributions

All authors listed, have made substantial, direct and intellectual contribution to the work, and approved it for publication.

## Funding

This work was supported by the following grants: NIH 8P20GM103436 and 1R15CA188890 (to MS), NIH R01DC014832 (to AG), and NIH R15DC013900 and an Action on Hearing Loss grant (to AC).

### Conflict of interest statement

The authors declare that the research was conducted in the absence of any commercial or financial relationships that could be construed as a potential conflict of interest.
